# An Updated Overview of the Magnetic Resonance Imaging of Brain Iron in Movement Disorders

**DOI:** 10.1155/2022/3972173

**Published:** 2022-02-24

**Authors:** Nicola Tambasco, Pasquale Nigro, Andrea Chiappiniello, Federico Paolini Paoletti, Sara Scialpi, Simone Simoni, Pietro Chiarini, Lucilla Parnetti

**Affiliations:** ^1^Movement Disorders Center, Neurology Department, Perugia General Hospital and University of Perugia, Perugia, Italy; ^2^Neurology Department, Perugia General Hospital and University of Perugia, Perugia, Italy; ^3^Department of Medical Physics, Perugia General Hospital, Perugia, Italy; ^4^Neuroradiological Unit, Perugia General Hospital, Perugia, Italy

## Abstract

Brain iron load is one of the most important neuropathological hallmarks in movement disorders. Specifically, the iron provides most of the paramagnetic metal signals in the brain and its accumulation seems to play a key role, although not completely explained, in the degeneration of the basal ganglia, as well as other brain structures. Moreover, iron distribution patterns have been implicated in depicting different movement disorders. This work reviewed current literature on Magnetic Resonance Imaging for Brain Iron Detection and Quantification (MRI-BIDQ) in neurodegenerative processes underlying movement disorders.

## 1. Introduction

In movement disorders, conventional magnetic resonance imaging (cMRI) is the most common and least invasive technique of neuroimaging. However, cMRI often does not allow for the detection pathological changes of disease [1, 2]. Several MRI techniques are sensitive to magnetic susceptibility (an intrinsic magnetic property of materials) variations induced by paramagnetic materials, which are able to detect subtle changes in several conditions [3]. Moreover, iron provides most of the paramagnetic metal signals in the brain, and their increases are observed in different neurodegenerative diseases, not only in neurodegeneration with brain iron accumulation (NBIA) syndrome, but also in other disorders including Huntington's disease (HD) [4] and Parkinson's disease (PD) [5, 6].

The iron deposition in the corresponding area of neuronal cell loss and astrocytic gliosis does not necessarily indicate that it plays a causative role but however might consist in an epiphenomenon of altered metal homeostasis [7]. On the other hand, an increase in toxic' iron may also enable the oxidative stress that underlies progression of disease [8]—neurons are highly susceptible to free radical-mediated injury due to their requirement for high levels of oxidative metabolism, and the fact that neuronal membranes are rich in polyunsaturated fatty acids [9].

Current literature is incomplete regarding the role of iron accumulation in the brain and its effects. This work reviewed current literature on Magnetic Resonance Imaging for Brain Iron Detection and Quantification (MRI-BIDQ) in neurodegenerative processes underlying movement disorders.

## 2. MRI for Brain Iron Detection and Quantification (MRI-BIDQ)

Different MRI techniques are currently available for investigation and detection of brain iron patterns, of which, some are intended to generate iron-weighted contrasts (qualitative assessment), whereas others have been designed to quantify iron concentrations and distributions [10].

**Figure 1:**
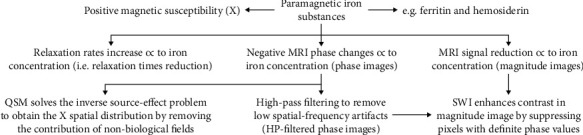
Iron detection related to MRI techniques.

The interaction between water molecules and paramagnetic iron complexes, such as ferritin and hemosiderin, contribute to the transversal magnetization dephasing [11]. Being so, T2-weighted spin-echo and gradient echo (GRE), as well as T2^∗^-weighted GRE MRI techniques, have been extensively used in the study of deep gray matter nuclei in order to evaluate the presence of iron accumulation [12]. Consequently, the paramagnetic iron causes a progressive signal reduction in T2 and T2^∗^-weighted MRI, proportionally to its concentration [13] (Figure 1).

Additionally, other works have focused on assessing the relaxation rates R2 (1/T2) and R2^∗^ (1/T2^∗^), which in many cases seem to be linearly related to iron concentration [10]: at the same time, relaxation rates, such as T2 and T2^∗^ relaxation times, are known to be influenced not only by iron deposition but also by other diamagnetic and paramagnetic deposits, as well as water changes across the tissues; since water content in brain tissue interferes on the relationship between iron and R2/R2^∗^ parameters [14].

Phase images are considered a direct measure of the variations of magnetic susceptibility (*Δχ*), which can be defined as the magnetic response of a substance when this response is placed in an external magnetic field: a specific property of each substance, which influences the local magnetic field values, in accordance with the following relation:

(1) $$\begin{array}{c} \Delta B=cV\Delta \chi B_{0}, \end{array}$$

where *c* is the concentration of the substance, *V* is the volume of the voxel, and *B*_0_ is the applied magnetic field [15]. Paramagnetic substances have a positive susceptibility *χ* and strengthen the magnetic field, whereas diamagnetic substances, such as calcium phosphate, present a negative *χ*, and weaken the local magnetic field. Phase information is mathematically regulated by the relation:

(2) $$\begin{array}{c} \Phi \left(\text{phase}\right)=-ɣ\Delta Bt, \end{array}$$

where *ɣ* is the gyromagnetic constant, which is equal to 2*π*∙42.58 MHz/T for protons, Δ*B* represents the induced magnetic field variation, and *t* is the time at which the signal is measured (usually the echo time TE from a gradient echo sequence) [6]. Thus, paramagnetic iron generates a negative phase value (*Φ*). In particular, the more iron is present in a tissue, the more phase values of that tissue decrease. The high-pass (HP) filters can be applied to phase images to remove low spatial-frequency artefacts (HP-filtered phase images), principally related to the static magnetic field inhomogeneities [16].

**Figure 2:**
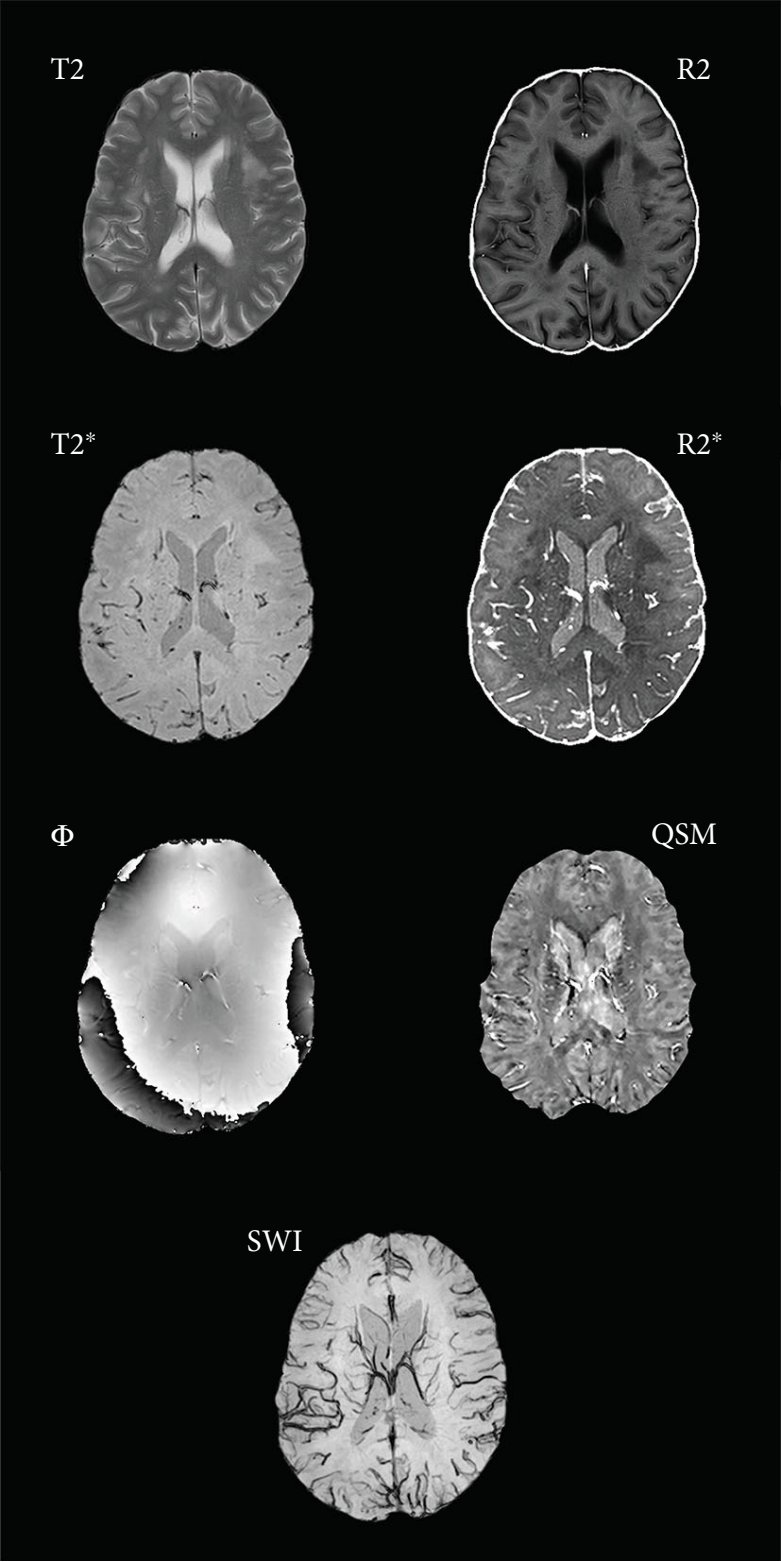
Rendering of axial sequence comparison in the same patient; iron distribution is much more detected by T2^∗^, R2^∗^, SWI, and QSM.

Susceptibility-weighted imaging (SWI) provides an additional measure for detecting iron-related changes by combining magnitude and phase T2^∗^-weighted data into a single image [17]. The phase image is corrected by applying a HP filter to remove undesirable artefacts. A phase mask is created from the HP-filtered phase image to enhance the contrast in the magnitude image by suppressing pixels with definite phase values. To generate the final SWI image, the phase mask is multiplied with the magnitude several times until the desired contrast is obtained [16]. The SWI images resulted to be extremely sensitive in the detection of diamagnetic and paramagnetic substances [18, 19] (Figure 2).

A novel method to assess paramagnetic and diamagnetic substances is represented by quantitative susceptibility mapping (QSM) [20], that is, an advanced MRI postprocessing technique solving the inverse source-effect problem to quantify local tissue magnetic susceptibility from the major magnetic field distribution (Figure 1). QSM extracts the spatial distribution of magnetic susceptibility from T2^∗^-weighted MRI phase or local field data, by removing the signal contribution of the non-biological background field ,that is, an advanced MRI postprocessing technique solving the inverse source-effect problem to quantify local tissue magnetic susceptibility from the major magnetic field distribution (Figure 1). QSM extracts the spatial distribution of magnetic susceptibility from T2^∗^-weighted MRI phase or local field data, by removing the signal contribution of the nonbiological background field [21]. The QSM has proven to be an accurate method for estimating iron levels in vivo, by showing an increased susceptibility [22].

## 3. Materials and Methods

### 3.1. Search Strategy

A thorough literature search was conducted using the online database PubMed, by entering the key words “T2”, “T2^∗^”, “R2”, “R2^∗^”, “susceptibility weighted imaging”, “SWI”, “SWAN”, “quantitative susceptibility mapping”, and “QSM” from 1990 to 2020. We linked “Parkinson”, “parkinsonisms”, “Huntington”, “chorea”, “hemiballism”, “essential tremor”, “neurodegeneration with brain iron accumulation”, “NBIA”, Hallervorden-Spatz”, “tic”, “Gilles de la Tourette”, “dystonia”, “Wilson”, and “aging”.

## 4. Results

### 4.1. MRI-BIDQ in Normal Aging

The brain iron accumulation is not only a pathologic event but it also represents a physiological process leading to a higher iron content in some brain structures throughout life. In 1958, Hallgren and Sourander characterized iron content in the deep grey matter at different ages [23]. A correlation between age and decreased signal intensity indicating a higher iron deposition in the basal ganglia (BG) has been reported recently in SWI. In fact, iron deposition increases in the putamen, white frontal matter, and red nucleus (RN) significantly from 22 to 70 years, differently from globus pallidum (GP), substantia nigra (SN), and caudate nucleus (CN) that do not increase strongly over the same period of life, suggesting that there is a rapid iron increase in these structures over the first two decades and a slower increase afterwards [24, 25]. MR field-dependent relaxivity increase (FDRI) estimates the transverse relaxation change across field strengths. FDRI has been reported to be more specific than SWI in detecting age-dependent accumulation of nonheme brain iron [13] (Table 1).

### 4.2. MRI-BIDQ in Essential Tremor

Although the pathophysiology of essential tremor (ET) remains poorly understood [26], recent investigations have provided evidence of increased iron accumulation in anatomical regions that are critically associated with ET. Moreover, GP, SN, and right dentate nucleus have revealed differences in T2^∗^ signals, when compared to controls whereas R2^∗^ values of the GP have further supported these findings [27]. Conversely, an analysis of nigral R2^∗^ did not show significant different values between controls and ET patients, suggesting a normal iron load [28] (Table 1).

The SWI, and in particular the detection of the nigrosome-1 area, has proven to be of aid in the differentiation between ET and PD patients, with high sensitivity and specificity [29]. A visual analysis combining neuromelanin-sensitive magnetic resonance imaging (NM-MRI) and nigrosome-1 imaging using QSM in SN has also shown a greater iron deposition in PD than ET [30].

Regarding surgical options for treating medication-refractory symptoms, these include thalamotomy or deep brain stimulation (DBS), which is able to improve symptomatology from 50% to 90% [31]. Hence, the in vivo visualization of the anatomical areas targeted by DBS has been the focus of numerous studies. Although the subthalamic nucleus (STN) is currently the preferred structure for DBS, the placement of an electrode in the zona incerta (ZI) offers greater therapeutic benefit in suppressing tremor in both PD and ET patients. Specifically, in two recent studies, the ZI was best visualized with T2^∗^-FLASH2D sequences by 3.0 T [32] and 7 T MRI [33]. Additionally, the detection of neurovascular structures with DBS planning in patients with different kinds of movement disorders has had significantly higher sensitivity on SWI when compared to T1-Gd enhanced MRI [34].

### 4.3. MRI-BIDQ in Choreic Disorders

#### 4.3.1. Huntington's Disease

HD is a genetic neuropsychiatric disorder that causes behavioral, cognitive, and motor dysfunction [35]. The pathological cascade of events in HD is complex and not fully understood. Transition metals, particularly iron, have been reported a role in its pathogenesis [4]. Both in vivo and ex vivo findings support the hypothesis of iron excess in the brain of HD patients, although there is no current evidence implicating early increases in brain iron as a trigger of the pathological process [36]. Histological reports have described the profound cellular structure deteriorations of the putamen and CN [37], as well as iron accumulation [38, 39].

Regarding the assessment of iron accumulation, various different techniques are available including T2, R2, and R2^∗^ relaxometry, magnetic field correlation (MFC), FDRI, SWI, and QSM. Particularly, T2 hypointensities in the BG have been associated with higher Unified Huntington's Disease Rating Scale (UHDRS) values, higher CAG numbers, and greater probabilities of developing symptoms within 5 years in gene carriers; suggesting that T2 hypointensities in the BG might be a biomarker for HD [40]. Moreover, increased brain iron in the GP (not in the putamen or CN) of pre-manifest patients (pre-HD) has been reported, suggesting that this iron accumulation might start long before disease onset.

Using R2 relaxometry, an increased iron deposition in the GP has been reported [41]. Moreover, in a multimodal approach with T1/T2/R2 measurements, it has been observed that increased iron in BG was independent of aging and started before any clinical manifestation of HD [42]; all the patients evidenced increased ferritin in their BGs, particularly in the GP during early disease stage. Moreover, an increase in R2^∗^, as well as atrophy in both CN and putamen, have been reported, suggesting that susceptibility values in these structures are inversely correlated with structure volume and directly correlated with genetic testing [43].

Using MFC values, increased levels of iron deposition in various brain structures have been observed [7, 44], without any observed differences between pre-HD and controls. In fact, iron accumulation has been revealed in both CN and putamen of patients with early HD vs. both controls and pre-HD. These findings are in contrast with those from Vyzimal et al. [41] and Jurgens et al. [40], but in line with those obtained by Bartzokis et al. [45]. The latter author reported on an increase in the FDRI signals in the CN, putamen, and GP of HD patients. An extension of this study [46] evidenced decreased signals in both the frontal white matter and genu of the corpus callosum.

For SWI, pre-HD, and HD showed progressive increases in the phase evolution of the GP, CN, and putamen, associated with increased disease severity, beginning in pre-HD long before the presence of clinical symptoms and increasing with proximity to the expected onset. Advanced HD patients have even shown higher field mapping values in the cortex [47]. Moreover, a hypointense signal of the GP bilaterally together with a milder hypointensity at the borders of the putamen and the CN in two cases of young onset HD has been reported [48].

QSM evaluation has been used in a cross-sectional investigation, showing significantly increased iron deposition in the GP and CN, both in pre-HD and HD, compared with controls. Moreover, a significant positive correlation between iron deposition increase in both putamen and CN, and disease burden score has been found [49]. Furthermore, van Bergen et al. demonstrated, by QSM, an increase of iron levels in the CN, putamen, and GP of pre-HD subjects [43] (Table 1).

In conclusion, whether or not, iron deposition is found elevated in the BG the structures seem to be moderately dependent on the technique used. Conceivably, the changing form of iron present in the different structures might be responsible for the variance in the reported results. Finally, whether one considers iron to be elevated in pre-HD might plausibly depend on an accurate interpretation of the clinical cut-off points [36].

#### 4.3.2. Chorea-Acanthocytosis

Chorea-acanthocytosis (ChAc) is a rare hereditary disorder characterized by involuntary choreiform movements and erythrocytic acanthocytosis [50]. The MRI in ChAc is typically reported as resembling HD: marked atrophy of the CN and putamen, a lesser extent of the cortex, an increased signal in the atrophic striatum on T2-weighted imaging, and rarely white matter abnormalities in the periventricular area bilaterally [51]. In a single case, an increased iron level by SWI has been observed in the corresponding area of T2 hyperintensity [52] (Table 1).

#### 4.3.3. Hemichorea-Hemiballism

Hemichorea-hemiballism (HCHB) is defined as a unilateral, involuntary, random movement disorder and secondary to lesions in the contralateral BG. Nonketotic hyperglycemia is a rarer cause for this presentation [53], especially in elderly patients with poorly controlled diabetes mellitus. The characteristic imaging sign is the striatal hyperintensity on T1-weighted images with no signal abnormality on T2, fluid attenuation inversion recovery (FLAIR), GRE, or DWI [54].

The pathophysiological basis of T1 shortening remains unclear. Puneet Mittal, describing SWI findings in a case of HCHB, excluded hemorrhage as the etiology of HCHB syndrome, based on the disproportionate extensive hyperintensity on the initial T1W sequence with comparatively little SWI hypointensity. The presence of ipsilateral prominent cortical veins supported the transient vascular insult on the ipsilateral side [55]. Similarly, Dharsono et al., in a single case observed over 5 months, an improvement of hyperintensity on T1-MRI and a more extensive and increased SWI hypointensity within the affected corpus striatum, suggesting an ongoing process of deposition of paramagnetic material. Iron-deposition-related neurotoxicity could explain the progressive malacic change demonstrated on follow-up imaging [56] (Table 1). Cherian et al. suggested a paramagnetic mineral deposition in the affected putamen caused by swollen gemistocytes that express metallothionein and zinc secondary to ischemic insult [57]. A recent accepted theory of HCHB is that of hypoperfusion due to hyperviscosity of blood because of hyperglycemia, which could enhance anaerobic metabolism leading to reduced GABA levels and increased thalamocortical activity [58]. Ohara et al. described autopsy-proven lacunar infarcts associated with reactive astrocytosis within the affected putamen [59], while Neal et al. described the mineral deposition in hypertrophied astrocytes located within the ischemic brain [60].

### 4.4. MRI-BIDQ in Degenerative Parkinsonisms

#### 4.4.1. Parkinson's Disease

In PD, SN is one of the main brain regions which is early on affected by the neurodegenerative process. The anatomical alterations of SN can be detected using T2^∗^-weighted GE sequences on 7 T MRI, which can show changes in the boundaries between SN and the crus cerebri, including a loss of the SN's smooth surface with its lateral and anterior profiles, replaced by an undulated aspect predominating in the more severely affected side, located in the rostral region [61]. In fact, Cho et al. have suggested that the loss of these smooth and clear arch-like boundaries might serve as a diagnostic marker [62]. Utilizing 7 T MRI, T2^∗^ nigrosomal hyperintensity is not always visible in PD patients [61, 63]. Furthermore, T2^∗^-weighted and neuromelanin sensitive sequences have detected a hypointense signal in the pars compacta of the substantia nigra (SNc) [64], and studies localizing SNpc with neuromelanin-sensitive contrast have evidenced PD related iron changes, particularly in its lateral-ventral part [65]. Finally, the overlap between the iron content, determined by R2^∗^ mapping, and neuromelanin in the SNpc, has been proposed as a neuroimaging biomarker for diagnosing PD [66]. By means of T2 and T2^∗^-MRI, increased iron contents in the GP, CN and slightly more so, in the SNc have been reported [67]. The association between SN iron load and clinical features has been recently explored: SN iron load has been correlated positively with disease duration and UPDRS-III off score; Montreal Cognitive Assessment, Spatial Span, and Graded Naming Test scores have all been reported to be inversely associated with SN iron accumulation, whereas, Wechsler Adult Intelligence Scale-IV Similarities score has been reported to have an inverse relationship with iron load in the putamen, GP, CN, RN, SN, dentate nucleus, and frontal white matter [68]. A correlation with the severity of PD motor impairment has also been observed by quantitative R2^∗^ in SN and GP [69].

Regarding SWI Signal Intensity (SWI-SI), PD patients had significant differences in SN compared to controls. The absence of the lateral “bright spot” in the SN has also been reported [70]. Specifically, the “swallow tail” appearance, characterized by hyperintensity in the dorsolateral SN on axial SWI, is characterized as a unilateral or bilateral loss [71, 72]. Instead, the absence of “swallow tail” sign in PD patients more the often corresponds to a reduction of nigrosome-1 and a loss of its signal intensity [71]; despite any increase iron deposition, probably caused by different tissue alterations, including neuromelanin loss, changes in iron oxidation state, or dopaminergic cell degeneration [66], confirmed on (123) I-FP-CIT SPECT [73]. With disease progression, the loss of hyperintensity also tends to extend to the nigrosome-4 [74]. On SWI, the absence of nigrosome-1's typical droplet-like high signal may serve as a marker for PD given its high sensitivity and specificity [75]. Likewise, the absence of dorsolateral nigral hyperintensity (DNH) on SWI-like images can reach up to 97% [76]. In fact, several SWI studies have produced conflicting results concerning disease progression: the loss of DNH on 3 T SWI in patients at H&Y stages I–II and III–IV [72]. Additionally, DNH loss is missing in at least two-thirds of the subjects presenting iRBD, it might be a predictor of prodromal PD [77], given that it is known that iRBD patients commonly have ipsilateral deficiency of the dopamine transporter [78]. Nevertheless, whenever the swallow-tail sign has marginal diagnostic accuracy in discriminating PD from atypical parkinsonism on 3 T SWI sequences [79], the detection of nigrosome-1 could be a marker for differentiating idiopathic PD and atypical progressive parkinsonism from controls [80].

Additionally, a loss of the trilaminar organization (a central hyperintense layer between two hypointense laminae) has been reported in SN in PD patients utilizing 7.0 GRE 3D SWI, due to its high sensitivity, specificity, positive predictive value, and negative predictive value [70].

Regarding the pathological undulated aspect of the SN lateral and anterior profile, detected by T2^∗^-weighted MRI, it cannot be confirmed by SWI and, according to Cosottini et al., it is not suitable for diagnosing PD, since SN changes in PD do not involve the reticular component. Moreover, the anterior border of the pars reticulata belonging to the substantia nigra (SNr) cannot be precisely identified, as extends beyond its anterior anatomic landmark [70, 81]. Finally, in the medial SNc, SWI has been reported to have a lower signal intensity compared to controls [69], even at 2-year follow-up [67].

Using SWI, the corrected phase (CP) values of the SN are generally significantly low in PD patients [82]. With regard to phase shift values, which correlate positively with iron concentration, these are significantly higher in the SN of PD patients, compared to controls [83], therein suggesting a significant increase in the most affected side [84]. In fact, the SNc in PD patients has been reported to have lower phase radian values, compared with controls [85]. These values have been found to have a positive correlation with disease severity, applying the UPDRS motor score as along with the bradykinesia-rigidity subscore [84]. Furthermore, the average phase values for bilateral SN can have a strong inverse correlation with the UPDRS motor score in those patients having akinetic/rigidity-predominant symptoms [86].

CN and RN have also been correlated with low SWI phase radian values when compared with healthy controls [85]. Moreover, differences in the anterior GP have been reported in patients with postural instability [69]. Moreover, the corrected phase values of RN and putamen also have been reported to be significantly decreased in PD patients, showing bilateral symmetry in iron deposition [82]. Whereas, Liu et al., using corrected phase values, did not observe in CN and GP significant differences between PD patients and controls [87]. Likewise, a 3 T SWI study reported no significant relationship between the UPDRS motor score and overall signal intensities of RN, putamen, GP, head of the CN, and thalamus. While in the same study, SWI hypointensity in the putamen was significantly correlated with the obtained Tinetti total score [88].

A significant increase in SN susceptibility on 3.0 T enhanced T2^∗^-weighted angiography scanning (ESWAN) has been reported in PD patients, both in tremor dominant and akinetic/rigid variants [89]. In fact, the ESWAN is a 3D multi-echo gradient-echo pulse sequence with partial flow compensation, using multiple magnitude or phase images with different echo times for image generation: the first echo applies the arterial inflow effect, whereas longer echoes are responsible for susceptibility effects. Comparing 3.0 T and 7.0 T acquisitions on high-resolution 3D-SWAN, it has been reported that the typical alterations of the SN in PD patients are evident at 7.0 T [90], which was probably due to an increase in the magnetic susceptibility effects of paramagnetic substances caused by a higher magnetic field [18]. Moreover, using ESWAN sequences, a potential association between the severity of PD motor symptoms and iron concentration in the regions of interest has been suggested [91].

QSM can reveal an increased susceptibility in the SN of PD patients when compared to controls [24]. When adopting 3D texture analysis, the QSM significantly outperformed R2^∗^ in this task [89]. In fact, the iron distribution patterns varied between PD patients and healthy controls, and the rates of abnormal deposition started diverging as early as the age 43 [92]. In PD, iron deposition has been reported to be high in the inferior part of the SN compared to both the middle and the superior parts. In healthy individuals, the middle and inferior part is similarly affected, being the superior part of the SN the least affected by iron accumulation. Regarding the SNc, iron distribution increases from the superior to the inferior part, both in PD and controls [93]. Moreover, SNc is predominantly altered in the early stages of disease, while SNr is involved the later stages [92]. Thomas et al. reported on QSM increases covarying with lower MoCA scores in the hippocampus and thalamus, poorer visual function, higher dementia risk scores in the parietal-frontal-medial occipital cortices, as well as higher UPDRS-III scores in the putamen [94]. Finally, a positive correlation between iron accumulation in the inferior parts of the SN and disease severity, as measured by PDQ-39, has been reported [93]. The QSM contrast images, when compared to T2^∗^-weighted sequences, offer an improved visualization of STN, both in PD and controls [95], assuring an accurate definition of the borders. STN connectivity has also been strongly negatively associated with a strong negative correlation with the QSM intensity of the thalamus, premotor, motor, and sensory regions, and a strong positive correlation for frontal, putamen, and brain stem areas [96] (Table 1).

All the reported findings suggest that iron-sensitive sequences a reliable tool for differentiating PD from controls (T2, T2^∗^, R2^∗^, SWI, ESWAN, and QSM), even at early stages (SWI and QSM). In addition, associations between BG iron load and overall motor features (T2^∗^, R2^∗^, ESWAN, and QSM), cognitive impairment (T2^∗^and QSM), and quality of life (QSM) have been reported, suggesting that iron-sensitive sequences can be effectively utilized for monitoring disease progression.

#### 4.4.2. Atypical Parkinsonism: Progressive Supranuclear Palsy and Multiple System Atrophy

Although conventional MRI provides signs considered as neuroradiological hallmarks of atypical parkinsonism, such as the “hummingbird” sign for PSP and the “hot cross bun” sign for MSA-c, none of these signs can be considered specific of any parkinsonian syndrome [97–100]. GRE and FLAIR sequences have been used in differentiating MSA from PD: the mean result on T2^∗^ GE sequences is a signal loss of the dorsolateral putamen in MSA patients, with a reported specificity of 91%, and the additional presence of a hyperintense lateral rim on FLAIR sequences has been shown to enhance the specificity up to 97% [101]. Additionally, SWI is able to improve the diagnostic accuracy of conventional 3.0 T MRI sequences in the work-up of parkinsonian syndromes. Particularly, SWI increases the diagnostic accuracy in MSA, with an increase of sensitivity up to 50% (and a preservation of high specificity) [102]. The support vector machine (SVM), an automated analysis of SWI, has been reported to improve diagnostic performance in the discrimination between PD and atypical parkinsonisms with an accuracy of around 90% [103]. On 3.0 T SWI, unilateral absence of DNH has revealed a high sensitivity and specificity for PD, MSA, and PSP; when bilateral, it achieved a high sensitivity and specificity of 100% [72]. Consequently, the loss of DNH is presently considered a neuroradiological marker not only for PD but also for MSA and PSP as well. Recent studies have reported the absence of the “swallow tail” sign even in dementia with Lewy bodies (DLB) [104]. That is, “swallow-tail” sign scores, obtained by SWI, appeared to be lower in idiopathic PD than in MSA [105]. However, the absence of DNH evaluated by SWI seemed to be unable to distinguish among the different neurodegenerative parkinsonisms [72].

Other than SN, utilizing SWI, different BG has been investigated, mostly using the hypointensity score as the primary measure. Specifically, a voxel-based analysis was carried out on iron-related SWI signals, where the detected hypointense signal was able to differentiate not only between PD and atypical parkinsonism but also between different types of atypical parkinsonism. Its presence, however, in the anterior putamen was able to differentiate the following: PSP from PD in the anterior and medial GP, PSP from MSA-p in the anterior and medial thalamus, and PSP from MSA-p along with PD and controls [106]. Moreover, in PSP, the hypointensity score of the RN on SWI has been observed to be significantly higher, when compared to MSA-p, PD, and controls; a score ≥ 2 is able to distinguish PSP from MSA-p and PD [107]. Several studies have confirmed the importance of the SWI-SI of the RN in discriminating PSP from other parkinsonian disorders [102, 106]. SWI-SI of the putamen seems to be the most reliable tool for discriminating MSA-p from other parkinsonian syndromes. Compared to PD patients and healthy subjects, patients with MSA-p have been reported to have a marked signal hypointensity and higher phase-shift values in the putamen, especially on the contralateral side of the most symptomatic side [108]. A lateral to medial gradient of the SWI-SI, resulting in putaminal hypointensity with a posterolateral hyperintense rim is suggestive of a very specific sign of MSA-p [109]. Given the importance of the SWI-SI of the putamen in recognizing MSA-p, different putaminal subregions have been investigated. When the putamen has been divided into 4 regions (upper outer, upper inner, lower outer, and lower inner), the lower inner region resulted being the most promising area for differentiating MSA-p from PD, using high iron percentage and total iron deposition as parameters [83]. Moreover, when ROIs were placed in the anterior and posterior regions of the putamen, respectively, the posterior region resulted being the most sensitive area for discriminating MSA-p from PD [110]. Furthermore, in MSA-p patients, hypointense SWI-SI, prevalent in the posterolateral putamen, has been able to differentiate MSA-p from PSP; even in the posterolateral area of GP, hypointense SWI-SI was higher in MSA-p. Patterns of iron deposition are different between MSA-p and PSP: in MSA-p, hypointensity is prevalent in the posterolateral regions of the putamen and GP, while in PSP, it is prevalent in the anteromedial areas of the same nuclei [106]. Signal intensity ratio (SIR) could help differentiate atypical parkinsonism from healthy controls and PD in the putamen. When considering RN, SIR appears lower in PSP than in MSA-p, PD, and healthy controls [111]. The putaminal hypointensity in MSA-p has been seen to be even present in those patients with a disease duration of <1 year, but, to date, no SWI studies have reported a significant relationship between neuroradiological parameters and demographic or clinical features, such as patient age, disease duration, or severity of disease, expressed by UPDRS-III and H&Y [109].

Finally, utilizing QSM, different topographical patterns of brain iron accumulation have been reported. An increase in susceptibility values in the RN and GP resulted higher in PSP when compared to PD, MSA, and controls. Differently, putaminal susceptibility values have appeared to be higher in MSA than in both PD and controls [24] (Table 1).

### 4.5. MRI-BIDQ in Idiopathic Adult-Onset Focal Dystonias

Idiopathic adult-onset focal dystonias are rare disorders, where one region of the body is affected by involuntary, sustained muscle contractions that cause twisting movements and abnormal postures. To date, only two studies have investigated iron content in these patients and their results were conflicting: both examining patients with idiopathic cervical dystonia. Aschermann et al. reported an increased R2^∗^ relaxation rate in the GP, suggesting an increased iron content [112], whereas a more recent multimodal quantitative MRI study (T1, T2, T2^∗^, and proton density) comparing patients with idiopathic cervical dystonia and healthy controls did not reveal significant group differences [113] (Table 1). Currently, available results support a common view that idiopathic cervical dystonia might resemble a functional network disease.

### 4.6. MRI-BIDQ in Wilson's Disease

Wilson's disease (WD) is an autosomal recessive inherited disorder characterized by low ceruloplasmin serum levels and copper accumulation, particularly in the liver and brain. Moreover, high iron deposition levels play an important role in this neurodegenerative process [114]. The accumulation of both copper and iron, two paramagnetic elements that shorten T2 relaxation time [115, 116], determines a hypointense signal in the BG, particularly in the GP, detectable on T2 and T2^∗^-weighted imaging, as well as on SWI [117]. On T2-weighted imaging, a high signal of the deep grey nuclei, due to pathological alterations, such as edema, gliosis, and neuron depletion, can mask the hypointense signal, due to the accumulation of paramagnetic iron and copper. Thus, SWI is regarded as outperforming traditional MRI sequences considering its ability to quantify the neurodegenerative process related to iron accumulation in WD [118–120]. Several brain regions, such as the putamen, have been found to have low signal intensities on SWI, whereas they present high-signal intensities on T2-weighted imaging in the same patients with WD. SWI hypointensity signal appears to be most prominent in the anterior lentiform nucleus, with the aspect of multiple concentric dark foci [121]. Moreover, SWI hypointensity has been observed even in the superficial layers of the cerebral cortex, especially in precentral, postcentral, and occipito-temporal gyri [122]. On SWI, patients with WD have had significantly lower phase values in the bilateral putamen (the most strongly affected area), bilateral head of the CN, thalamus, RN, SN, and GP when compared to controls [123]. CP values in the right CN and left putamen are lower in cerebral type compared to hepatic type WD patients [124]. Overall, this finding seems to be in line with the temporal course of the pathological process, which affects the early liver and subsequently the brain. Whenever neurologic disorders occur in patients, copper and iron contents are higher than those in patients with only hepatic symptoms [118]. No correlation has been reported between the asymmetry of CP values in the subcortical nuclei and the motor asymmetry [125]. Furthermore, a negative correlation has been reported between SWI phase values of GP and the severity of dysarthria, while a negative correlation has been detected between SWI phase values of CN and the extent of tremor [120] (Table 1).

### 4.7. MRI-BIDQ in Neurodegeneration with Brain Iron Accumulation

Many neurodegenerative disorders, sharing a common profile of iron accumulation in the BG and associated with cognitive and movement dysfunction, as well as causative genetic mutations, are referred to as NBIA disorders, also known as Hallervorden-Spatz syndrome (HSS) [126]. Two of these nine different NBIA genetic mutations involve iron metabolism, while the remaining involve fatty acid metabolism or lysosomal activity [127].

#### 4.7.1. Pantothenate Kinase-Associated Neurodegeneration

The more relevant form of NBIA is pantothenate kinase-associated neurodegeneration (PKAN), caused by PANK2 gene mutations [128], resulting in dystonia, dementia, dysphagia, spasticity, rigidity, and tremor: typically, with onset during childhood. The MRI detection of iron deposition seems to precede the development of clinical symptoms [129]. Initially, GPs are symmetrically T2-hyperintense, a nonspecific finding related to edema and tissue damage secondary to an accumulation of cysteine-containing neurotoxic compounds [130]. This leads to an increased iron load in physiologically iron-rich brain structures such as the GP. T2-weighted imaging can evidence a central region of hyperintensity in the GP, due to gliosis, with surrounding hypointensity, due to iron deposition, called eye-of-the-tiger sign, which is not a specific sign of PANK2 [131].

The SWI technique may identify iron accumulation earlier than conventional MRI [132]. Moreover, SWI sequence could be able to discriminate the different profiles of iron deposition in PKAN (iron deposition only in nigropallidal pathway from SN to GP) from other forms of NBIA (iron accumulation also observed in the RN, dentate nucleus, putamen, or CN in other forms of NBIA) [127], aiding in the most appropriate choice for molecular genetic testing [133]. Moreover, FDRI technique is more sensitive than SWI in the detection of brain iron accumulation of PKAN [134]. In 10 patients, with childhood onset, the magnetic susceptibility effect of iron has been reported to enhance in the form of a lower signal intensity on T2^∗^W gradient echo imaging (fast low-angle shot), when compared to conventional imaging. Likewise, the abnormal bilateral deposition of a paramagnetic substance in the striatonigral tract was observed in two patients who undergone BOLD-SWI, suggesting that the striatonigral pathways may have been involved earlier during the disease [135].

QSM has been used to quantify iron deposition in several different BG ROIs in order to differentiate between homozygous and heterozygous PANK2 mutations: heterozygous and asymptomatic PANK2 mutation carriers did not present higher brain iron concentrations than controls, while iron deposition 3 times higher in the GP, SN, and internal capsule of PKAN patients [136].

#### 4.7.2. Mitochondrial Membrane Protein-Associated Neurodegeneration and Beta-Propeller Protein-Associated Neurodegeneration

Only case reports have been described with SWI findings in patients with mitochondrial membrane protein-associated neurodegeneration (MPAN): a hypointensity in bilateral GP and SN has been found, suggesting increased mineral deposition [137, 138]. The same radiological alterations have been confirmed in 15 Turkish patients with adult-onset disease [139] and in a 3-year-old girl with beta-propeller protein-associated neurodegeneration (BPAN) [140].

## 5. Conclusion

The brain iron levels in movement disorder patients are currently assessed using iron-sensitive MRI sequences along with data processing techniques. Up until the 1990s, T2 and R2 sequences were mainly employed for this task; since then, other methods including the well-regarded SWI and even more so QSM are predominantly used by researchers. Elevated iron levels are more often recorded when investigating by specific MRI techniques. Moreover, the changing form of iron present in the different structures might be responsible for the variances in reported results to date. Iron accumulation seems to play a key role, although not thoroughly understood, in the degeneration of the BG, as well as other brain structures implicated in movement disorders. Overall, specific iron distribution patterns seem to depict movement disorders, encouraging the use of MRI-BIDQ, whenever possible, in their diagnostic assessment. Finally, increased iron load does not seem to reflect motor disability but does appear to correlate with nonmotor symptoms, such as cognitive abilities, as observed in Parkinson's disease, expanding the prospective purpose of MRI-BIDQ studies. Further studies are needed to support these aspects.

## Figures and Tables

**Table 1:** Literature review of iron detection in movement disorders.

Authors	Cohort		Primary contrast	Main findings
*Normal aging*				
Harder et al. [25]	134 HC		SWI	GP, PUT, RN, and SN: important reduction in SWI signal intensity in the first two decades of life PUT and GP: plateau during the fifth decade RN and SN: continuous and gradual declining intensity throughout life
Xu et al. [24]	78 HC		SWI	PUT, RN, and FWT: significant age-related iron accumulation
Pfefferbaum et al. [13]	23 HC		SWI FDRI	GP: iron concentration relevant regardless of age PUT: higher iron concentration with aging FDRI more specific than SWI in detecting age-dependent iron accumulation

*Essential tremor*				
Novellino et al. [27]	24 ET 25 HC		T2^∗^ R2^∗^	GP, SN and right DN of ET: differences in signals when compared to HC GP: the most involved nucleus in brain accumulation
Homayoon et al. [28]	40 PD 15 tremors in dystonia 25 ET 25 HC		R2^∗^	No significant differences in nigral R2^∗^ values between HC and ET
Perez et al. [29]	16 PD 16 ET		SWI	N1 area: useful in differential diagnosis between PD and ET
Jin et al. [30]	68 PD 25 ET 34 HC		NM QSM	Combining NM and N1 visualization in the SN may aid in differential diagnosis of PD and ET

*Choreic disorders*				
Dumas et al. [7]	27 early HD 22 pre-HD 25 HC		T2 ASE MFC	Early-stage HD patients (but not premanifest gene carriers): higher iron concentrations in CN and PUT
Macerollo et al. [48]	2 HD		SWI	GP: marked symmetrical hypointensity PUT and CN: milder hypointensity at the periphery
Rosas et al. [47]	28 pre-HD 34 HD 56 HC		SWI FM	GP, CN, and PUT: higher FM values in pre-HD and HD than HC Left superior frontal, left middle frontal cortex, left and right anterior cingulate, left and right paracentral, left and right precuneus, right cuneus, and left precentral: higher FM values in advanced HD patients
Bartzokis et al. [45]	11 HD 27 HC		FDRI	CN, PUT, and GP: signal increase in HD patients
Bartzokis et al. [46]	11 HD 27 HC		FDRI	FWM and genu of the CC: increased signal in HD patients HIPP, THA, and splenium of the CC: unchanged signal
Van Bergen et al. [43]	15 pre-HD 16 HC		QSM R2^∗^	Pre-HD compared to HC: higher susceptibility values in CN, PUT, and GP and decrease in SN and HIPP. Atrophy+increases in R2^∗^ signal in CN and PUT
Dominguez et al. [49]	31 pre-HD 32 HD 30 HC		QSM	GP, CN, and PUT: increased iron deposition in pre-HD and HD compared to HC Iron accumulation in PUT and CN associated with disease severity
Lee et al. [52]	1 ChAc		SWI	GP, CN, and PUT: decreased SI value
Cherian et al. [57]	1 HCHB		SWI	Controlateral PUT: decreased SI value
Dharsono et al. [56]	1 HCHB		SWI	Controlateral PUT and GP: decreased SI value
Mittal et al. [55]	1 HCHB		SWI	Controlateral PUT: decreased SI value
*Parkinson's disease*				
Rossi et al. [65]	32 PD 19 HC		T2 T2^∗^	PD after 2 years of follow-up: relaxation increased in GP, CN, and SNc and decreased in THA
Tambasco et al. [68]	32 PD 10 HC		T2^∗^	SN iron load positively correlated with disease duration and motor impairment, inversely with Montreal Cognitive Assessment, Spatial Span, and Graded Naming Test scores Iron content in PUT, GP, CN, RN, SN, DN, and FWM inversely correlated with Wechsler Adult Intelligence Scale-IV similarities
Kwon et al. [62]	10 PD 10 HC		T2^∗^	PD: not smooth boundary between SN and crus cerebri
Blazejewska et al. [64]	1 PD 2 HC		T2^∗^ NM	PD: absence of N1
Langley et al. [63]	28 PD 54 HC		T2^∗^ SWI	PD: higher T2^∗^ hypointense signal in SNpc compared to HC. The greatest increase in lateral ventral region of SNpc
Rossi et al. [69]	36 PD 21 HC		R2^∗^	SN, GP: disease-related changes reflecting motor impairment
Langley et al. [65]	28 PD 28 HC		R2^∗^	Increased mean R2^∗^ in PD as compared to controls
He et al. [66]	39 PD 33 HC		R2^∗^ NM	R2^∗^values in the whole SNpc, SNpc overlap volume, and SNpc overlap percentage were larger in PD SNpc overlap percentage positively correlated with disease duration
Bae et al. [73]	126 PD 11 MSA 11 PSP 26 HC 36 DC		SWI	The presence or absence of nigral hyperintensity accurately visualized in 112 PD, 7 MSA, and 11 PSP patients and 53 controls SWI and SPECT concordance rate: 86.2% Loss of nigral hyperintensity on SWI suggests nigrostriatal dopaminergic degeneration, as indicated by SPECT
Calloni et al. [80]	56 PD 18 PSP 3 MSA-C 9 MSA-P 16 DC 24 HC		SWI	N1: - PD vs. HCs: SE 96,43%, and SP 85% - APPs vs. HCs: SE 100% and SP 85% - PD vs. APPs: SE 96,43%, and SP 0% N1: valid tool to differentiate PD-APPs from controls. Midbrain atrophy+PUT hypointensity: more accurate for distinguishing APPs from PD
Cosottini et al. [70]	17 PD 13 HC		SWI	PD: absence of the lateral bright spot of the SN
De Marzi et al. [77]	15 iRBD 42 HC 104 PD		SWI	Absence of DNH could identify prodromal degenerative parkinsonism in iRBD (loss in at least 2/3 of iRBD)
Gao et al. [75]	54 PD 51 N-PD 11 UD		SWI	The absence of typical droplet-like or oval-shaped N1 signals useful in identifying PD with high SE and SP
Jin et al. [86]	87 PD 50 HC		SWI	Nigral iron deposition is a risk factor in PD across multiple motor phenotypic expressions
Liu et al. [87]	60 PD 30 HC		SWI	PD: CP value of SN significantly decreased compared to HC. CP values of CN, PUT, and GP were not significantly different
Mahlknecht et al. [78]	364 PD 231 HC		SWI	Absence of dorsolateral nigral hyperintensity predicting ipsilateral dopamine transporter deficiency: SE 87.5% and SP 83.6%
Meijer et al. [79]	39 PD 21 AP		SWI	Swallow-tail sign: marginal diagnostic accuracy to discriminate between PD and AP
Qiao et al. [82]	30 PD 30 HC		SWI	PD vs. HC: significant differences in the CP values of SN, RN, and PUT (PD: low signal); no differences in other ROIs (GP)
Schneider et al. [88]	21 PD 19 PIGD		SWI	In PIGD lower intensity values in SN, PUT, GP, head of CN, THA compared to PD (especially PUT, GP). Positive correlation of PUT hypointensity with Tinetti total score in PD and PIGD
Schwarz et al. [71]	10 PD 9 HC	9 PD 81 HC	SWI	Subjects classified into PD and non-PD according to absence or presence of N1: in retrospective cohort SE 100%, SP 95%, NPV 1, PPV 0.69, and accuracy 96%
Sung et al. [74]	128 PD 15 HC		SWI	Early-stage PD: loss of hyperintensity observed more often in the N1 region. Intact SNs (both in N1 and N4): 9.6% of early-stage PD patients (not found in any of the late-stage PD patients)
Ide et al. [141]	20 PD 50 HC		SWI PADRE T2	Delineation of MML of GP in HC: good in 84% of cases on PADRE, in 34% of cases on SWI, and no delineation on T2 Delineation of MML in PD: good in 90% of cases on PADRE
Sugiyama et al. [76]	39 PD 8 PSP 13 MSA 34 HC		SWI PADRE	PADRE: obscuration of the boundary between crural fibers and SN, overall correct classification for neurodegenerative parkinsonism = 74% SWI: absence of dorsolateral nigral hyperintensity, PD = 97%, PSP = 100%, MSA = 67%, and HC = 6%. Overall correct classification = 96%
Martin-Bastida et al. [84]	70 PD 20 HC		Phase mapping	Phase radians: PD > HCs bilaterally in PUT, GP, and SN SN contralateral to the most affected side > less affected side SN radians positively correlated with UPDRS-III and bradykinesia-rigidity subscores
Zhang et al. [85]	42 PD 30 HC		Phase mapping	Phase radians of SNc, CN, and RN in PD patients lower than those in HC
Zhang et al. [142]	40 PD 26 HC		Phase mapping	Higher phase shift values in PD compared to HC in SN
Nam et al. [143]	6 PD 15 HC		SMWI	SMWI enhances the visibility of N1 when compared to conventional susceptibility contrast images
Cosottini et al. [90]	14 PD 13 HC		ESWAN	7 T images differentiating HC from PD with a higher diagnostic accuracy
Guan et al. [89]	62 PD 40 HC		ESWAN	Iron accumulation: - In SN: PD > HCs - In DN and RN: correlated with tremor symptoms - In CN: a potential markers for akinetic/rigid progression
Ji et al. [91]	54 PD 28 HC		ESWAN	Iron concentrations reflecting the severity of the PD motor symptoms
Alkemade et al. [95]	12 PD 12 HC		QSM T2^∗^	QSM: higher interrater agreement Contrast-to-noise ratios for QSM lower than T2^∗^
Li et al. [144]	28 PD 28 HC		QSM R2^∗^	QSM texture features successfully distinguishing PD from HC and significantly outperforming R2^∗^ texture analysis
Dimov et al. [96]	9 PD		QSM	STN connectivity and QSM intensity: - Strong negative correlation: THA, premotor, motor, and sensory regions - Strong positive correlation: frontal, PUT, brain stem areas
Guan et al. [93]	90 PD 38 HC		QSM	Iron distribution in SN: HC: middle part ≈ inferior part > superior part PD: inferior part > middle part > superior part Positive correlation between accumulation in inferior SN and PDQ-39
Kim et al. [145]	38 PD 25 HC		QSM	Susceptibility values significantly differed between PD and HC
Sethi et al. [92]	20 PD 174 HC		QSM	Abnormal iron load in SN of PD patients with greater volumes compared to HC
Thomas et al. [94]	100 PD 37 HC		QSM	In PD, QSM increases in the prefrontal cortex and PUT. QSM increases covarying with lower MoCA scores in the HiPP and THA, with poorer visual function and with higher dementia risk scores in parietal-frontal-medial occipital cortices, with higher UPDRS-III scores in the PUT

*Atypical Parkinsonisms*				
von Lewinski et al. [101]	88 PD 52 MSA 29 HC		T2^∗^	MSA: signal loss of the dorsolateral putamen in T2^∗^ (SP > 0.91, SE 0.64-0.69). When combined with hyperintense lateral rim in FLAIR sequences SP 0.97
Gupta et al. [107]	11 PD 12 PSP 12 MSA-p 11 HC		SWI	RN: HS > in PSP compared to other groups PUT: HS > in PSP compared to PD SN: HS > in PSP compared to other groups
Haller et al. [103]	16 PD 20 AP		SWI	Group-level comparison THA: higher values in PD compared to AP bilaterally SN: higher values in PD compared to AP (left side)
Lee et al. [109]	30 PD 11 MSA-p 30 HC		SWI	MSA-p: compared to PD and HC increased HS in PUT
Meijer et al. [110]	38 PD 3 PSP 12 MSA-p 3 DLB 13 HC		SWI	MSA-p: lower SI of PUT (bilaterally) compared to PD lower SI of CN (left side) compared to PD PSP: lower SI of RN and DN (left side) compared to PD and HC
Reiter et al. [74]	104 PD 22 PSP 22 MSA 42 HC		SWI	Unilateral absence of DNH: SE 94.7% and SP 97.2% to detect neurodegenerative parkinsonism Bilateral absence of DNH: SE 92.4% and SP 100% to detect neurodegenerative parkinsonism
Sakurai et al. [111]	13 MSA-p 12 PSP 12 PD 13 HC		SWI	*SIR* PUT: MSA-P < PSP and HC RN: PSP < HC–PD-MSA-p
Wang et al. [83]	16 PD 8 MSA-p 44 HC		SWI	MSA-p: increased iron deposition in PUT compared to PD Lower inner region of the PUT: valuable subregion to differentiate MSA-p from PD
Wang et al. [105]	39 MSA 18 PD 31 HC		SWI	PD: swallow-tail sign scores lower than MSA and HC. PUT hypointensity lower than MSA HC: PUT hypointensity lower than MSA
Yoon et al. [110]	30 PD 17 MSA-p		SWI	MSA-p: lower SI value in posterior PUT compared to PD Correlation between PUT low SI and (18)F-FDG PET hypometabolism
Hwang et al. [108]	50 PD 27 MSA-p 27 HC		SWI Phase mapping	MSA-p: compared to PD increased HS and phase shift values
Han et al. [106]	15 PD 11 PSP 12 MSA-p 20 HC		Phase mapping	Phase shift value, ROI-based analysis PSP: (vs. HC) higher in RN, SN, PUT, GP, and THA; (vs. PD) higher in RN, PUT, GP, and TH; (vs. MSA-p) higher in RN, SN, GP, and THA MSA-p: (vs. HC) higher in RN and PUT; (vs. PD) higher in RN and PUT PD: (vs. HC and vs. MSA-p) higher in SN Phase shift value, voxel-based analysis MSA-p: higher in the posterolateral PUT and GP PSP: higher in the anterior PUT, in the anterior and medial GP, in the anterior and medial THA
Sjöström et al. [24]	15 PSP 11 MSA 62 PD 14 HC		QSM	RN susceptibility: PSP > PD, PSP > MSA, and PSP > HC GP susceptibility: PSP > PD, PSP > MSA, and PSP > HC PUT susceptibility: MSA > PD and MSA > HC SN susceptibility: PD > HC

*Idiopathic adult-onset focal dystonias*				
Aschermann et al. [112]	12 CD 12 HC		R2^∗^	GP: increased R2^∗^ relaxation rate in CD
Gracien et al. [113]	17 CD 29 HC		T2 T2^∗^	No significant group differences

*Wilson's disease*				
Skowroñska et al. [117]	28 WD		T2^∗^ SWI	T2^∗^: GP hypointensity in 10 patients SWI: GP hypointensity in 20 patients
Cai et al. [146]	1 WD		SWI	Hyperintense area in the bilateral SN and lenticular nuclei
Lee et al. [121]	1 WD		SWI	Hypointense signal in SN, RN, lentiform nucleus, and CN
Lee et al. [122]	2 WD		SWI	Diffuse hypointense signal in the superficial layers of the cerebral, GP, SN, RN, and DN
Zhong et al. [119]	(76) 5 WD		SWI	Lenticular nucleus SWI hypointensity. Extensive hypointense lesions are visible only via SWI, not when using T2
Zhou et al. [120]	30 WD 20 HC		SWI	WD vs. HC: lower CP values in SN, right CN, and right GP
Zhou et al. [124]	40 WD		SWI	CP values of the right CN and left PUT in cerebral type WD patients lower than in hepatic type
Zhou et al. [125]	50 WD 20 HC		SWI	No correlation between the asymmetry of CP values in subcortical nuclei and the motor asymmetry in patients with WD
Bai et al. [118]	23 WD 23 HC		Phase mapping	In WD, compared to HC, lower negative phase shift values in bilateral PUT, bilateral CN, bilateral TH, bilateral RN, and bilateral SN. In neurological WD compared to hepatic WD, lower negative phase shift values in bilateral PUT and bilateral GP
Yang et al. [123]	33 WD 18 HC		Phase mapping	Lower mean phase values in the bilateral head of the CN, GP, PUT, THA, SN, and RN compared to the HC group. Bilaterally PUT is strongly affected

*NBIA*				
McNeill et al. [127]	26 PKAN		T2^∗^ T2	In a minority of PKAN cases: hypointensity of the dentate nuclei (1/5 on T2^∗^ sequences, 2/26 on T2)
Bosemani et al. [132]	1 PKAN		SWI	Low SI values in bilateral GP
Lee et al. [133]	1 PKAN		SWI	Low SI values in SN and GP
Vinod Desai et al. [135]	13 NBIA		SWI	Low SI values in striatonigral tract
Zhang et al. [147]	1 PKAN		SWI	Low SI value in GP
Dusek et al. [136]	2 PKAN 11 carriers 13 HC		QSM	Heterozygous PANK2 mutation carriers: no increased brain iron concentrations compared to HCs PKAN patients: higher concentrations of iron in GP, SN, and IC
Gore et al. [138]	1 MPAN		SWI	Hypointensity of the GP, caudate heads, and PUT and relatively hyperintense streaking of the medullary laminae
Olgiati et al. [139]	15 MPAN		SWI	Low SI bilaterally in GP and SN
Yoganathan et al. [137]	1 MPAN		SWI	Low SI values in SN and GP
Takano et al. [140]	1 BPAN		SWI	Low SI value in SN and GP
Roubertie et al. [148]	3 AP4M1		SWI	Low SI value in GP

HC = controls; DC = disease controls; HD = Huntington's disease; ChAC = chorea-acanthocytosis; HCHB = hemichorea-hemiballismus; PD = Parkinson's disease; N-PD: without PD; UD: suspected PD, undiagnosed; MSA = multiple system atrophy; MSA-p = multiple system atrophy, parkinsonian type; MSA-c = multiple system atrophy, cerebellar type; PSP = progressive supranuclear palsy; iRBD = idiopathic rapid eye movement sleep behavior disorder; DLB = dementia with Lewy bodies; AP/APP = atypical parkinsonism; CD = cervical dystonia; WD = Wilson's disease; PKAN = pantothenate kinase-associated neurodegeneration; NBIA = neurodegeneration with brain iron accumulation; PANK2 = pantothenate kinase 2; MPAN = mitochondrial membrane protein-associated neurodegeneration; BPAM = beta-propeller protein-associated neurodegeneration; FLAIR = fluid attenuation inversion recovery; SWI = susceptibility-weighted imaging; QSM = quantitative susceptibility mapping; NM = neuromelanin-sensitive; SMWI = susceptibility map-weighted imaging; ESWAN = enhanced T2^∗^-weighted angiography; PADRE = phase difference enhanced imaging; FDRI = field-dependent R2 increase; MFC = magnetic field correlation; FM = field map; FSE = fast spin echo; ASE = asymmetric spin echo; CP = corrected phase; SI = signal intensity; SIR = signal intensity ratio; HS = hypointensity score; DNH = dorsolateral nigral hyperintensity; SPECT = single-photon emission computed tomography; PUT = putamen; RN = red nucleus; GP = globus pallidus; SN = substantia nigra; SNpc/SNc = substantia nigra pars compacta; CN = caudate nucleus; HIPP = hippocampus; THA = thalamus; DN = dentate nucleus; STN = subthalamic nucleus; FWM = frontal white matter; IC = internal capsule; MML = medial medullary lamina; CC = corpus callosum; N1 = nigrosome 1; ROI = region of interest; CNR = contrast to noise ratio; UPDRS III = Unified Parkinson's Disease Rating Scale (Part III).

## Data Availability

No data were used to support this study.
